# Cystic Echinococcosis in Hospitalized Children from Western Romania: A 25-Year Retrospective Study

**DOI:** 10.3390/biomedicines12020281

**Published:** 2024-01-25

**Authors:** Ana Alexandra Paduraru, Maria Alina Lupu, Calin Marius Popoiu, Maria Corina Stanciulescu, Livius Tirnea, Eugen Sorin Boia, Tudor Rares Olariu

**Affiliations:** 1 Discipline of Parasitology, Department of Infectious Diseases, Victor Babes University of Medicine and Pharmacy, 300041 Timisoara, Romania; paduraru.ana@umft.ro (A.A.P.); lupu.alina@umft.ro (M.A.L.); liviustirnea@yahoo.com (L.T.); rolariu@umft.ro (T.R.O.); 2 Center for Diagnosis and Study of Parasitic Diseases, Department of Infectious Disease, Victor Babes University of Medicine and Pharmacy, 300041 Timisoara, Romania; 3 Patogen Preventia, 300124 Timisoara, Romania; 4 Clinical Laboratory, Municipal Clinical Emergency Hospital, 300254 Timisoara, Romania; 5 Clinical Laboratory, Institute of Cardiovascular Diseases, 300310 Timisoara, Romania; 6 Discipline of Pediatric Surgery and Orthopedics, Department of Pediatrics, Victor Babes University of Medicine and Pharmacy, 300041 Timisoara, Romania; mcpopoiu@yahoo.com (C.M.P.); stanciulescucorina@yahoo.com (M.C.S.); 7 Pediatric and Orthopedic Surgery Clinic, Emergency Clinical Hospital for Children “Louis Turcanu”, 300011 Timisoara, Romania

**Keywords:** hydatidosis, *Echinococcus granulosus*, zoonosis, parasitic disease, Romania

## Abstract

Cystic echinococcosis (CE) is a cosmopolitan parasitic disease caused by *Echinococcus granulosus.* We aimed to assess the epidemiological aspects of the disease in hospitalized children from Western Romania, a well-known endemic area for CE. We retrospectively investigated the medical records of children hospitalized between 1998 and 2022. A total of 144 patients were included, and 58.3% were from rural areas. The number of cases increased with age, from 9% in the age group 3–5 years to 59.7% in the age group 11–17 years. The liver was more frequently affected (65.3%), and a significant association between gender and the affected organ was noted; liver cysts were more frequently diagnosed in girls, while lung cysts were recorded mostly in boys. Complications were more frequently reported in patients with pulmonary CE compared to hepatic CE (*p* = 0.04). Boys had more complications (16/23, 69.6%) compared to girls (7/23, 30.4%) (*p* = 0.03). A third of the children were hospitalized for more than 14 days, and multiple hospitalizations were recorded in 31.3% of the patients. This paper provides new insights into the epidemiologic features of cystic echinococcosis in children from Western Romania. Our findings indicate that exposure to the parasite starts in childhood, and the rate of hospitalization increases with age. Public health strategies should be implemented and permanently improved in order to lower the prevalence of CE in children.

## 1. Introduction

Human cystic echinococcosis is a parasitic zoonotic infection caused by the larval stages of *Echinococcus granulosus sensu lato* (*s.l.*). Cystic echinococcosis is found on all continents except Antarctica [1]. According to recent estimates, 188,000 new CE cases are diagnosed each year, and the burden of the disease is 183,500 DALYs (disability-adjusted life years) worldwide [1]. About 10–20% of all CE cases are diagnosed in children [2,3].

The life cycle of *E. granulosus* involves a definitive host, canids, and intermediate hosts, ungulates [4]. Humans are accidental hosts and acquire the infection by ingesting the parasite’s eggs with contaminated water or food, through contact with dogs, or via the hand-to-mouth route [5]. After being ingested, the embryonated eggs release the oncospheres under the action of gastric fluids. In the small intestine, the hexacanth embryos attach to the enteric mucosa and penetrate it. They are subsequently transported to the various organs via capillaries’ blood flow [6]. The infection leads to the formation of one or more hydatid cysts in the organs [7]. Infection is often acquired during infancy or adolescence, and the incubation period is long, lasting many years [8]. Various patterns of hydatid cysts and multiple organ involvement have often been observed in children because of the undeveloped filtering mechanisms of the liver and lungs throughout infancy and childhood and the role of intestinal lymphatic vessels in parasite transmission [9].

Usually, cysts develop in the liver (70%) and lungs (20%), yet they can also be found in other organs like the brain, bones, heart, urinary, and genital tract [10,11]. However, in children, lungs are apparently more frequently affected because of the negative pressure, vascularization, and compressibility [12,13,14]. Symptomatology in the early stages of the disease is absent or mild [5]. When complications occur, the symptomatology depends on the stage, number, localization, and size of the cyst [5].

Pulmonary involvement can include nonspecific symptoms like dyspnea, cough, chest pain, and fever [15]. Pulmonary cysts are often discovered on a chest X-ray as an accidental finding or as a result of respiratory symptoms following cyst rupture or subsequent infection of the cyst. In children, pulmonary cysts have a wide differential diagnosis with simple cysts, inflammatory masses, and benign or malign tumors [15,16]. An accurate diagnosis requires multiple investigations, which include imaging, serology, histology, and the polymerase chain reaction (PCR) method [6,15].

When liver involvement happens, typically, cysts develop in the right lobe. Apart from a few exceptions, cystic lesions grow slowly and often simply turn symptomatic in older children or teenagers due to local consequences, leading to a sensation of pressure or discomfort in the right hypochondrium. Jaundice is a quite uncommon sign [10]. Liver hydatid cysts must be differentiated from hepato-renal fibrocystic disease and dysontogenic cysts [10].

In children with hydatid disease, in addition to the local action of the parasite, there is a significant release of acute phase inflammatory mediators, which are involved in the pathogenesis of the disease and also in the development of functional and organic lesions. This causes the suppression of the main humoral and cellular mediators, leading to hemodynamic changes, microcirculatory dysfunctions, and an imbalance of inflammatory mediators [17].

Imaging techniques are crucial assets not only for the diagnosis of CE but also for establishing the localization and the stage of the cyst. The World Health Organization Informal Working Group on Echinococcosis (WHO-IWGE) classified the hydatid cyst into three groups (“active”, “transitional”, and “inactive”) according to their stage. The “active” group includes unilocular cysts (CE1) and multi-vesicular cysts with daughter vesicles (CE2). The “transitional” group includes cysts characterized by endocyst detachment (CE3a) and mostly solid cysts with daughter vesicles (CE3b). The “inactive” group contains mostly non-viable cysts with involution and solidification of the content and a growing amount of calcification (CE4, CE5). The WHO-IWGE classification proved to be an important tool that helps in the management and follow-up of patients with CE. However, finding cysts with a diameter beneath 2 cm poses difficulties for imagery [18,19]. Enzyme-linked immunosorbent assays (ELISAs), used as screening tests, and immunoblotting (IB), utilized as a confirming assay due to their greater specificity and sensitivity, are now the major immunological testing procedures in CE patients [18]. Serology is unable to distinguish between exposure to *Echinococcus* and active disease [15]. Moreover, children’s immune systems seem to be less reactive to hydatid cysts, and antibody production is thought to be lower in children than in adults. As a result, serological tests for CE in children are more prone to provide false negative results [9]. *Echinococcus* species may be distinguished from one another and from other cestodes by using recently developed DNA-based techniques, such as quantitative and/or nested PCR analysis, which are sensitive and relatively specific [19].

Several factors, including educational, agricultural, cultural, socio-economical, and environmental conditions, play an important role in the transmission of the disease [20]. According to studies conducted on dogs from rural areas in North-western Romania, up to 34% of animals were found to be infected [21]. Large amounts of *Echinococcus* eggs may be easily transferred from animals to vegetables, agricultural fields, or into water, where they can remain infectious for months [8]. Contaminated water, soil, and crops raise the potential for animal and human infection [8]. Adults in rural areas typically work as farmers and livestock herders [8], and the disease is known to be prevalent in agricultural and pastoral regions [18,22].

Although CE may affect people of all ages, younger individuals seem to be more susceptible [20]. The presence of free-roaming dogs in places where children usually play, such as playgrounds, constantly exposes them to *Echinococcus* eggs [23]. Children from agricultural families are sometimes withdrawn from school so they can be involved in agricultural activities like shepherding and field watering. These duties require handling livestock animals and canines as well as working with egg-contaminated soil and plants [8,24]. It has been previously shown that children who spend their first five years of life in rural regions are more likely to acquire the infection compared to children who live the same period in urban areas and also to children who migrate from cities to rural areas after turning five [25].

Of the total Romanian population, 45.7% live in rural areas, and approximately 30% of the population is involved in the agricultural sector [26,27]. Moreover, it was demonstrated that lower education levels increased the chances of acquiring the infection [24]. Herders in rural regions often have only primary or secondary education [24]. In Romania, the rate of functional illiteracy in the last decade was between 40 and 45%, and the school dropout rate was around 15% [28].

Romania is regarded as a highly endemic country for cystic echinococcosis [29,30,31]. Recent studies demonstrated that this zoonotic infection is still present among people and animals [27,31,32,33,34,35]. In Romania, there are no formal national programs for the control of *E. granulosus*, and there is no requirement to report new cases. The only data source available to identify CE cases are hospital medical databases.

Few reports are available to the international scientific community regarding CE in children, and data regarding this zoonosis in Romanian children are limited. Therefore, we decided to assess the demographic features of cystic echinococcosis in hospitalized children from Western Romania for a period of 25 years.

## 2. Materials and Methods

### 2.1. Study Population

A retrospective study comprised the patients aged <18 years between 1 January 1998 and 31 December 2022 in Western Romania. Children were hospitalized with cystic echinococcosis in referral centers in children’s healthcare from Western Romania: Emergency Clinical Hospital for Children “Louis Turcanu” Timisoara and County Emergency Clinical Hospital Arad.

Hospital databases and medical charts were reviewed. Data regarding age, gender, area of residence, length of hospital stay, cyst location, and complications were collected and analyzed. Children were grouped into three categories according to their school age: 3–5 years (preschool), 6–10 years (elementary school), and 11–17 years (middle/high school).

According to the medical files, the diagnosis was established using imaging techniques (radiography, ultrasonography, computed tomography) and confirmed by anatomopathological investigation.

### 2.2. Study Area

The study was conducted in Arad County and Timis County, Western Romania. In 2022, the estimated population in the two counties was 1,224,186 inhabitants. Rural inhabitants accounted for 43.9% of the population in Arad County and 43.6% in Timis County. In the two counties, females accounted for 51.4% and 51.7% of the total population, respectively [36]. Timis County is the largest county in Romania, with 8696.7 km^2^, while Arad County encompasses an area of 7754 km^2^.

The Mediterranean influences and the temperate—continental climate in both counties result in warm summers and mild winters, which are ideal for the development of technological plants and grains. The geography is characterized by a predominant plain in both counties, favorable for sheep raising [37,38].

### 2.3. Statistical Analysis

Collected data were added to a Microsoft 365 Excel database, version 2205 (Microsoft Corp., Redmond, WA, USA). Statistical analyses were performed using the software packages MedCalc for Windows, version 20.015 (MedCalc Software, Ostend, Belgium) and EpiInfo (v.7.2, CDC, Atlanta, GA, USA, 2018). T-test and Chi-square test of independence, Fisher test, and Mantel–Haenszel test were used to assess the relationship between variables. A *p*-value < 0.05 was considered statistically significant.

The CE incidence was determined according to the yearly population estimates presented by the Romanian National Institute of Statistics [36].

This study was conducted in accordance with the Declaration of Helsinki and approved by the Victor Babes University Ethics Committee in Timisoara, Romania (No. 4 from 8 February 2018).

## 3. Results

A total of 144 patients <18 years were diagnosed and hospitalized with cystic echinococcosis in Western Romania between 1 January 1998 and 31 December 2022. Patients included in the study aged between 3 and 17 years (mean age 11.4 ± 3.8 years). Results indicate an age-related rate for CE diagnosis with an increase from 9% in children aged 3–5 years to 59.7% in children aged 11–17 years. Most of the subjects were residents of rural areas 84/144 (58.3%) (Table 1). We found no association between gender and area of residence (*p* = 0.59) or gender and age groups (*p* = 0.63). However, we found a significant association between areas of residence and age group distribution (χ^2^ = 6.37, *p* = 0.04). Most of the patients from the age group 11–17 were from rural areas (43/86, 50%) compared to children from the age group 6–10 years (14/45, 31.1%) (*p* = 0.04).

**Figure 1:**
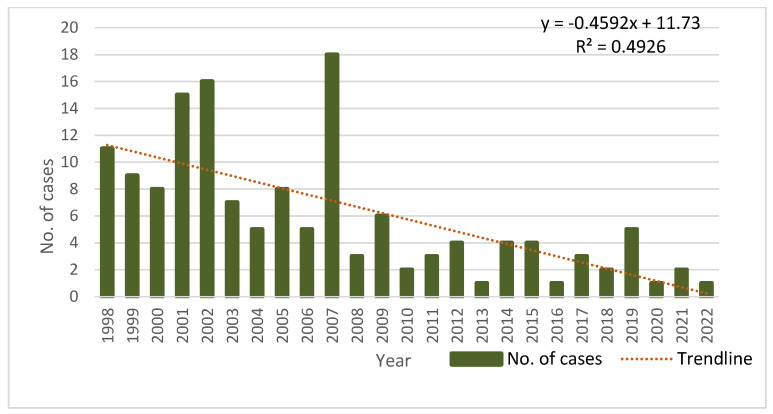
Distribution of CE cases in children from Western Romania between 1998 and 2022.

During the studied period, the number of cases varied between 1 and 18 cases/year, with 11 cases in 1998 and 1 case in 2022. Overall, a significant descending trend in the number of cases was observed (R^2^ = 0.492, *p* = 0.0001). The same outcome was observed when analyzing the trend of the incidence during the studied period (R^2^ = 0.42, *p* = 0.0004) (Figure 1).

The length of hospital stay varied from 1 to 50 days with a mean of 12 ± 9.1 days, with no statistically significant difference between boys (12.7 ± 10.2 days) and girls (11.2 ± 7.9 days) (*p* = 0.32). The mean length of hospital stay in patients with complications was 14.3 ± 9.3 days compared to patients without complications, 11.5 ± 9 days (*p* = 0.19). Fifty-three (36.8%) of the 144 children were hospitalized for up to 7 days, and 42 (29.2%) between 8 and 14 days. Interestingly, 49/144 (34%) of the children were hospitalized for more than 14 days, and 45 (31.3%) of the patients had multiple hospital presentations. Of the 45 patients, 34 (75.6%) had the same organ involvement.

The most affected organ was the liver (94/144, 65.3%), followed by the lungs (43/144, 29.8%), spleen (2/144, 1.4%), kidneys (2/144, 1.4%), peritoneum (2/144, 1.4%) and pancreas (1/144, 0.7%) (Table 2). We observed a significant association between gender and the organ affected (χ^2^ = 9.8, *p* = 0.007). In liver involvement, girls were more frequently affected (56/94, 59.6%), while in lung involvement (27/43, 62.8%) (*p* = 0.02) and other localizations (6/7, 85.7%) (*p* = 0.04), boys were more affected.

Multiple organ involvement was observed in 20/144 (13.9%) cases. The liver was associated with lungs in 12/20 (60%) cases, with the spleen in 3/20 (15%), and in 3/20 (15%) with other organs (small intestine, gallbladder, kidney). Lung hydatid cysts were associated with muscle (1/20, 5%) involvement and with kidney (1/20, 5%) involvement. The presence of multiple cysts in the same organ was reported in 23/144 (16%) cases. Seventeen (73.9%) of the 23 children had multiple cysts in the liver, 4/23 (17.4%) in the lungs, 1/23 (4.3%) in the kidney, and 1/23 (4.3%) in the peritoneum.

Complications were reported in 23/144 (16%) cases. Most reported complications were cyst infection (11/23, 47.8%) and fistulization (3/23, 13%). One patient experienced acute pancreatitis with superinfection of the cyst (1/23, 4.3%). Other complications (8/23, 34.8%) were recorded as allergic reactions, pleural effusion, pneumothorax, arrhythmia, and hemoptysis. Complications were present in 11/43 (25.6%) patients with pulmonary involvement compared to 11 (11.7%) of 94 patients with hepatic involvement (*p* = 0.04). Complications were more frequently reported in boys (16/23, 69.6%) compared to girls (7/23, 30.4%) (*p* = 0.03).

## 4. Discussion

Cystic echinococcosis is a cosmopolitan parasitic disease [5], which, in developing countries, continues to be a public health problem [39]. Standard of living, medical awareness, and the efficacy of veterinary care are the social factors that may impact the disease. The size and density of the dog population, the presence of parasitic eggs in the environment, the rate of parasitic infection among intermediate hosts, and the disease’s potential of transmission between the definitive and intermediate hosts are environmental factors favoring the disease prevalence [40]. Illegal animal slaughter, poor abattoir facilities in rural regions, and the negligence of slaughterhouse staff members who dispose of contaminated viscera by giving them to stray dogs all contribute to the maintenance of CE transmission [8]. A previous study demonstrated that dog ownership in a household with children is highly linked with CE, no matter if the dog is owned in the present or was owned up to 10 years ago. Even more, the risk of CE tends to increase with the number of dogs owned [25].

In Romania, CE was reported in animals, including sheep, swine, and cattle. Between 1998 and 2003, in slaughterhouses from Timis District, prevalences of 22.36%, 5.83%, and 4.32% were reported in cattle, sheep, and swine, respectively [41]. In northeastern and southern Romania, prevalences of 49.87% in sheep and 32.34% in cattle were identified between 2009 and 2011 [31]. Darabus et al. [42] described a CE rate of 2.45% in cattle in a recent study conducted between 2020 and 2021. This decrease could be a sign that Romania’s sanitary-veterinary control measures have improved at the farm level. The possibility for veterinarians to advise owners on deworming has also increased because of the advancements in canine population control programs like the microchipping of dogs [42]. It is also important to highlight that the extent of sheep-raising activities, particularly the density of sheep in the area, has been associated with the prevalence of human CE. This is additionally associated with the rates of human CE-related hospital admissions [43].

Although the disease can affect people of all ages, about 10–20% of all CE cases are diagnosed in children [2,8]. It has been hypothesized that in regions where CE prevalence in adults is elevated, the disease rate is also high in children [44]. Because agricultural workers’ homes are close to the fields, children are frequently seen playing in the fields and coming in contact with contaminated soil. This type of exposure increases the risk of young people acquiring the infection due to negligence while playing or due to pica disorder, leading to direct hand-to-mouth transmission [8].

Results of the present study suggest that exposure to the parasite starts from childhood, and the rate of hospitalization may increase with age. Similar to our findings, Dashti et al. [12], Çelebi et al. [45], and Kaman et al. [46] reported the mean ages of children with CE to be 11.5 ± 6.1 years, 12.4 years, and 10.3 ± 3.5 years, respectively. More than half of our study participants were registered as children aged 11–17 years. Huamán et al. [47], Salazar-Mesones et al. [48], and Talaiezadeh et al. [49] found a higher number of cases in the age group 5–9 (90/177, 50.8%), 6–11 (37/55, 67.3%), and the age group 6–10 (46/76, 60.5%), indicating an early exposure to the parasite [48]. Preschoolers and young elementary school children are more susceptible to CE due to poor hygiene standards and close contact with dogs [50]. Moreover, the prevalence of CE in children indicates recent trends in disease transmission in endemic areas and could be a vital indicator for assessing the effectiveness of CE management programs [9].

Our findings also revealed a higher prevalence in rural areas. It has been previously shown that CE is most frequently encountered in rural areas [4,27]. The disease is usually prevalent in rural areas with agricultural activities, regional climate, low socioeconomic levels, and improper animal slaughter [51]. In rural regions, major sources of infection might arise from contaminated water supplies, vegetables irrigated with contaminated water, or vegetables contaminated with dogs’ feces [8]. Moreover, the percentage of cases from urban areas may be overestimated due to patients coming from surrounding peri-urban regions or having a regular history of traveling to rural high-risk zones [8].

Interestingly, we did not observe differences in the number of cases between boys and girls. Previous studies demonstrated that boys from rural areas seemed to be more exposed to infection because they met the outside environment usually earlier than girls [6,52,53]. On the other side, Moroni et al. and Kosenglu et al. reported more cases of CE in girls [54,55].

Our results indicate a decreasing tendency in the number of cases and in the incidence of CE in children from Western Romania through the 25-year study period. This trend can be attributed to the socio-economic conditions in the territory (change in the way of working in the rural area, the aging of the rural population) or to higher disease awareness among community members and among medical professionals [27,56]. Stronger norms in the areas of safety, food, and health have been implemented after Romania joined the European Union (EU) in 2007, which may be another factor contributing to the downward trend [57]. Similar results were also observed by Amahmid et al. [8] and Pierangeli et al. [58]. In Morocco, a decline was noted in the incidence of CE in children from 5.3/100,000 to 1.7/100,000 inhabitants during the 7-year study period (2010–2016) [8]. In Patagonia, Argentina, the incidence decreased from 22.1/100,000 in 1995 to 6.2/100,000 in 2004 [58]. In Greece, an incidence of 0.08/100,000 [6] inhabitants was reported, while in Turkey, the incidence in children reached up to 150 cases/100,000 inhabitants [59]. However, it is important to note that the COVID-19 pandemic might have played a role in the notable reduction in CE cases between 2020 and 2022, when hospital admissions were limited.

Regarding the mean hospital stay, Mfingwana et al. [15] reported 9 ± 5.4 days as the average length of stay, longer in patients with complications (12.5 ± 6.6 days) compared to those without complications (6.8 ± 1.5 days) (*p* < 0.001). In our study group, the length of hospital stay did not vary significantly because of complications. Interestingly, we observed that a third of the children were hospitalized for more than 2 weeks, and almost a third of the children experienced multiple hospital presentations.

More than half of the patients had liver involvement. Similar results were reported by Djuricic et al. [60] and Kaman et al. [46]. Other studies demonstrated that the lung, rather than the liver, is the most common location for hydatid cysts in children [12,14,47]. Vascularization, negative pressure, and elasticity of the tissue seem to favor the development of cysts in the lung [13,14,61]. Another reason for the higher number of pulmonary cases could be the earlier and louder symptoms of lung cysts observed in children (e.g., cough, chest discomfort), which sometimes push parents to seek medical treatment more quickly [8].

Of the patients with lung involvement, almost two-thirds of our study participants were boys. Of the patients with liver involvement, more than half were girls (*p* = 0.02). As previously shown, boys are more likely to develop lung hydatid cysts than females, who are more likely to have liver hydatid cysts [62]. It has been hypothesized that biological or cultural factors may be responsible for this association [63]. According to the literature, hydatid cyst prevalence in unusual anatomic sites (other than the liver and lungs) is similar in children and adults, ranging from 7% to 13% [60]. In Serbia, the percentage of hydatid cysts in uncommon anatomic locations in children was 9.2% cases [60]. In this study, the presence of hydatid cysts in uncommon anatomical locations was observed in 7/144 (4.9%) cases. Of note, one in seven children hospitalized in Western Romania had multiple organ involvement. Similar outcomes were reported in Ethiopia and Iran, with rates of 11.8% and 15.2%, while a higher rate was reported in South Africa (38.1%) [53].

One in six of our study participants had complications recorded as cyst superinfection and/or fistulization. This is higher than the rate of complications reported in Thessaloniki, Greece (7.48%) but lower than those described in Ethiopia, where more than half of the children hospitalized with CE had complicated cysts [6,53]. Cyst rupture was also described as a complication by Aygun et al. [64] and Dashti et al. [12] in 12 (21.6%) and 7 (12.3%) cases. The infection most often arises from locations around the hydatid cyst (such as the bronchial or biliary tree) or as a side effect of bacteremia from any source. By compressing and distorting the biliary tree, the cyst increases its chances of getting infected [65]. However, it is believed that in order to become infected, the endocyst and pericyst must be ruptured [66]. Rupture of the cyst may be spontaneous, during surgery or trauma [51]. Occasionally, cysts may burst into the bronchus, causing severe hemoptysis [67]. Intrabronchial rupture might be accompanied by severe dyspnea, sometimes resulting in asphyxia and death [68].

There are certain study limitations that should be taken into account. First, the study’s retrospective design. Our results are based on the available information obtained from patients’ medical records. The charts did not provide details on the morphological characteristics of the cysts, nor did they provide information on additional epidemiological elements that could be potential risk factors for the disease. Second, the database only included patients who were hospitalized with CE in two hospitals in Western Romania. The data may not accurately represent the total number of humans with CE in the two counties, which could be higher because many patients may be asymptomatic and remain unnoticed. It has been previously demonstrated that CE antibodies may be detected in asymptomatic individuals, including blood donors [32,69,70].

## 5. Conclusions

Our study provides new insights into the epidemiologic features of cystic echinococcosis in pediatric settings. Although we observed a decreasing trend of CE cases, the disease is still a significant public health problem in Western Romania. The presence of CE among children indicates that exposure to the parasite occurs during childhood.

Reference hospitals should develop interdisciplinary teams to improve the diagnosis and treatment of CE patients. Implementation of the WHO-IWGE classification of cysts according to their viability should be implemented to facilitate case management. Moreover, developing a surveillance system that requires the reporting of all new cases could help in evaluating disease trends and improve patient follow-up.

This research demonstrates the need for better strategies to prevent disease transmission. Public health strategies should be implemented and improved. Because the majority of infections occur at a young age, educational campaigns for echinococcosis control should be started with students in elementary school. Unhealthy behaviors (lack of hand hygiene, eating fruits or vegetables without washing, drinking water from an unknown source) that originate in childhood are likely to persist during adulthood; as a result, changing children’s behavior through health education programs has the potential to significantly reduce incorrect health behaviors later in life. Health education is a critical tool and an effective method for achieving short- to long-term management of CE.

Moreover, effective communication between pediatricians, family physicians, and educational professionals is recommended to increase awareness about the disease and find solutions to prevent it. The use of digital learning toolkits, comic video cartoons, and other interactive teaching techniques could facilitate children’s learning about CE. The results of the current study should serve as a foundation for monitoring the evolution of this disease, which may aid in the development of control methods.

## Figures and Tables

**Table 1:** Demographic characteristics in children hospitalized with cystic echinococcosis in Western Romania between 1998 and 2022.

Variables		No. Cases (%)
Age groups	3–5	13 (9)
	6–10	45 (31.3)
	11–17	86 (59.7)
Gender	Female	73 (50.7)
	Male	71 (49.3)
Area of residence	Rural	84 (58.3)
	Urban	60 (41.7)

**Table 2:** Distribution of CE cases according to the cyst localization.

Variables	Liver (%)	Lung (%)	Other (%) *	Total (%)
Gender				
Female	56 (59.6)	16 (37)	1 (14.3)	73 (50.7)
Male	38 (40.4)	27 (63)	6 (85.7)	71 (49.3)
Total	94 (65.3)	43 (29.8)	7 (4.9)	144 (100)

* Other: spleen (2 cases), kidney (2 cases), peritoneum (2 cases), pancreas (1 case).

## Data Availability

Data are available upon request.
